# Suctioning of clear amniotic fluid at birth: A systematic review^☆^

**DOI:** 10.1016/j.resplu.2022.100298

**Published:** 2022-09-17

**Authors:** Joe Fawke, Jonathan Wyllie, Enrique Udaeta, Mario Rüdiger, Hege Ersdal, Mary-Doug Wright, Myra H. Wyckoff, Helen G. Liley, Yacob Rabi, Gary M. Weiner, International Liaison Committee On Resuscitation Neonatal Life Support Task Force

**Affiliations:** a Department of Neonatology, University Hospitals Leicester NHS Trust, Leicester, UK; b Department of Neonatology, James Cook University Hospital NHS Trust, Middlesbrough, UK; c Committe of Neonatology, Mexican Association of Pediatrics, Mexico; d Saxony Center for Feto-Neonatal Health, Medizinische Fakultät, TU Dresden, Dresden, Germany; e Critical Care and Anaesthesiology Research Group, Stavanger University Hospital, Norway; f Faculty of Health Sciences, University of Stavanger, Norway; g Apex Information, Vancouver, Canada; h Division of Neonatal-Perinatal Medicine, Department of Pediatrics, The University of Texas Southwestern Medical Center, Dallas, TX, United States; i Mater Research Institute and Mater Clinical School, Faculty of Medicine, The University of Queensland, Brisbane, Australia; j Department of Pediatrics, University of Calgary, Calgary, Alberta, Canada; k Division of Neonatal-Perinatal Medicine, C.S. Mott Children's Hospital, University of Michigan, Ann Arbor, MI, United States

**Keywords:** Neonatal resuscitation, Airway, Suctioning, Basic life support, Bpm, beats per minute, CI, confidence interval, CoE, certainty of evidence, DR, delivery room, GRADE, Grading of Recommendations, Assessment, Development and Evaluation, ILCOR, International Liaison Committee on Resuscitation, IQR, interquartile range, MD, mean difference, NICU, neonatal intensive care unit, NLS, Neonatal Life Support, NNT, number needed to treat, PICO, population, intervention, comparison, outcome, PPV, positive pressure ventilation, PRISMA, Preferred Reporting Items for Systematic Reviews and Meta-analyses, Quasi-RCT, quasi-randomized controlled trial, RCT, randomized controlled trial, RD, risk difference, RoB, risk of bias, RR, risk ratio, SGA, supraglottic airway device, SR, systematic review

## Abstract

**Context:**

Upper airway suctioning at birth was considered standard procedure and is still commonly practiced. Negative effects could exceed benefits of suction.

**Question:**

In infants born through clear amniotic fluid (P) does suctioning of the mouth and nose (I) vs no suctioning (C) improve outcomes (O).

**Data sources:**

Information specialist conducted literature search (12th September 2021, re-run 17th June 2022) using Medline, Embase, Cochrane Databases, Database of Abstracts of Reviews of Effects, and CINAHL. RCTs, non-RCTs and observational studies with a defined selection strategy were included. Unpublished studies, reviews, editorials, animal and manikin studies were excluded.

**Data extraction:**

Two authors independently extracted data, risk of bias was assessed using the Cochrane ROB2 and ROBINS-I tools. Certainty of evidence was assed using the GRADE framework. Review Manager was used to analyse data and GRADEPro to develop summary of evidence tables. Meta-analyses were performed if ≥2 RCTs were available.

**Outcomes:**

Primary: assisted ventilation. Secondary: advanced resuscitation, oxygen supplementation, adverse effects of suctioning, unanticipated NICU admission.

**Results:**

Nine RCTs (n = 1096) and 2 observational studies (n = 418) were identified. Two RCTs (n = 280) with data concerns were excluded post-hoc. Meta-analysis of 3 RCTs, (n = 702) showed no difference in primary outcome. Two RCTs (n = 200) and 2 prospective observational studies (n = 418) found lower oxygen saturations in first 10 minutes of life with suctioning. Two RCTs (n = 200) showed suctioned newborns took longer to achieve target saturations.

**Limitations:**

Certainty of evidence was low or very low for all outcomes. Most studies selected healthy newborns limiting generalisability and insufficient data was available for planned subgroup analyses.

**Conclusions:**

Despite low certainty evidence, this review suggests no clinical benefit from suctioning clear amniotic fluid from infants following birth, with some evidence suggesting a resulting desaturation. These finding support current guideline recommendations that this practice is not used as a routine step in birth.

**Funding:**

The International Liaison Committee on Resuscitation provided access to software platforms, an information specialist and teleconferencing.

**Clinical Trial Registration:**

This systematic review was registered with the Prospective Register of Systematic Reviews (https://www.crd.york.ac.uk/prospero/) (identifier: CRD42021286258).

## Introduction

At birth, all infants have fluid-filled lungs and upper airways. Lung fluid is absorbed within the lungs. Healthy infants may clear upper airway fluid by some combination of swallowing, inhalation and sometimes, sneezing. Despite this, longstanding practice was to routinely provide oro/nasopharyngeal suctioning at birth in many parts of the world. There have been increasing concerns that this practice may not confer benefit and may have undesirable consequences.

ILCOR prepared an evidence worksheet in 2010 and concluded that: “*Routine intrapartum oropharyngeal and nasopharyngeal suctioning for newborn infants with clear or meconium-stained amniotic fluid is no longer recommended*”.^1^

The World Health Organisation (WHO) reviewed 3 studies^3^, ^4^, ^5^ in a 2017 systematic review^6^ and recommended that: “*In neonates born through clear amniotic fluid who start breathing on their own after birth, suctioning of the mouth and nose should not be performed.*”. The WHO guideline authors made a further consensus-based recommendation that: “*In neonates born through clear amniotic fluid who do not start breathing after thorough drying and rubbing the back 2*–*3 times, suctioning of the mouth and nose should not be done routinely before initiating positive pressure ventilation. Suctioning should be done only if the mouth or nose is full of secretions.*”.

In addition to no benefit, both ILCOR and WHO found literature suggesting possible adverse effects of suctioning, including lower oxygen saturations over the first 10 minutes of life and lower likelihood of Apgar score of 10 at 10 minutes. Other reported associations include increased risk for bradycardia^4^, ^7^, ^8^ apnea,^8^ hypoxemia and arterial oxygen desaturation,^3^, ^9^, ^10^ hypercapnia,^11^ impaired cerebral blood flow regulation,^12^ increased intracranial pressure^13^ and infection.^8^

One study reported that suctioning was commonly applied despite opposing recommendations in resuscitation guidelines.^14^

This question was prioritized by the ILCOR Neonatal Life Support Task Force because an ILCOR scoping review in 2019 found sufficient new studies to justify updating the systematic review,^2^ and to assess the certainty of evidence using Grading of Recommendations, Assessment, Development and Evaluation (GRADE) methodology.^15^ The aim of the review was to assess the role of routine suctioning of clear fluid in the upper airway, compared to no routine suctioning in newborn infants.

## Methods

### Protocol

This systematic review (SR) was completed as part of the ILCOR NLS Task Force continuous evidence review process based on knowledge gaps identified in the 2020 ILCOR NLS Consensus on the Science of Resuscitation with Treatment Recommendations.^2^ The SR and meta-analysis were guided by the Cochrane Handbook for Systematic Reviews of Interventions^15^ and reported following the Preferred Reporting Items for Systematic Reviews and Meta-Analyses (PRISMA) statement for meta-analysis of health care interventions.^16^ The protocol was registered with the Prospective Register of Systematic Reviews (PROSPERO; CRD42021286258) on 22nd October 2021. The study was conducted in the a priori planned way included in the Prospero registration, except for updated literature search dates where database access was subtly different.

### Inclusion and exclusion criteria

Randomized controlled trials (RCTs) and non-randomized studies (non-randomized controlled trials, interrupted time series, controlled before-and-after studies, cohort studies) are eligible for inclusion. Unpublished studies (e.g., conference abstracts, trial protocols), review articles, editorials, comments, case reports, animal studies, and manikin studies were excluded. All years were included without language restrictions if an English abstract was available.

For this review, observational studies were cohort studies eligible for inclusion if they used a defined strategy to ensure that the participants were either all of those who received an exposure of interest in a defined population (e.g., infants born at a hospital between specified dates), or they were sampled in such a way as to be representative of such a population. Otherwise, the study was an (ineligible) case series.

### Population, Intervention, Comparator, Outcome, Study Design, Time Frame (PICOST) question

Among neonates who are born through clear amniotic fluid in the delivery room (population) does initial suctioning of the mouth and nose (intervention) compared with no initial suctioning (comparison) change outcome?

The PICOST question was developed by the authors in collaboration with the ILCOR NLS Task Force and approved by the ILCOR Scientific Advisory Committee.

Outcome ratings using the GRADE certainty of evidence (COE) classifications^17^ of critical or important outcomes were based on a consensus for international neonatal resuscitation guidelines (range 1–3 low importance, 4–6 important but not critical, 7–9 critical for decision-making).

The primary outcome was receipt of assisted ventilation (important). Secondary outcomes were advanced resuscitation (critical), receipt and duration of oxygen supplementation (important), adverse effects of intervention (important) and unanticipated admission to the Neonatal Intensive Care Unit (NICU) (important). Appendix A defines these outcomes.

Sub-group analyses were defined a priori as gestation age categories (≥34 + 0, 28 + 0 – 33 + 6, <28 + 0 weeks), route and method of delivery (vaginal vs caesarean section), suction device used (bulb or suction).

### Search strategy

Literature searches in Medline, Embase, the Cochrane Database of Systematic Reviews, the Cochrane Central Register of Controlled Trials, the Cochrane Methodology Register, the Database of Abstracts of Reviews of Effects, and Cumulative Index to Nursing and Allied Health Literature (CINAHL) were developed by an information specialist (MDW) iteratively, in consultation with the review team. The subject headings and keywords were adapted for the respective databases. The search was completed on 12th September 2021 and updated on 17th June 2022. For the updated literature search the EBM Review suite of databases was no longer available through the Information Specialist’s institution. In order to recreate the original search, the Cochrane Library (online through Wiley) was searched for CDSR and CCRCT (Trials). Covidence Systematic Review software^18^ was used for management of the search results.

### Study selection

Authors independently screened titles and abstracts, studies required agreement from two authors to be excluded or included for full text review. Full text reviews were conducted independently by authors and two authors need to agree on inclusion. Disagreements were resolved by consensus of the full review team. The process was conducted using Covidence software (Veritas Health Innovation, Melbourne, Australia).

### Data extraction, bias, and quality assessment

The study review group worked collaboratively to extract data from included studies. Study investigators were emailed if data queries arose. All data for pre-specified outcomes were included where studies reported on these outcomes. Studies were assessed for risk of bias (RoB) using the Cochrane ROB2 tool^19^ for RCTs and the Risk of Bias in Non-Randomized Studies of Interventions (ROBINS-I)^20^ for observational studies, using templates constructed in Covidence systematic review software.^21^ RoB was defined at a study level, and where studies contributed data to an individual outcome, their RoB for that outcome was assessed. All RoB assessments were decided by consensus of the full review group.

Certainty of evidence (confidence in the estimate of effect) for each outcome was decided by consensus among the review group using the GRADE framework.

Review team members were excluded from assessing inclusion or RoB for any study in which they had participated as an investigator. The evidence profile tables were presented and discussed with the ILCOR NLS Task Force and content experts.

### Data analysis

Review Manager^22^ was used to analyse data and GRADEPro^23^ to develop summary of evidence tables. Meta-analyses were performed if ≥2 RCTs were available. Observational studies were analysed and reported if fewer than 2 RCTs were available. For dichotomous outcomes, pooled unadjusted risk ratios (RRs) and corresponding 95% confidence intervals (CIs) were reported using the Mantel-Haenszel fixed effect method. The pooled risk difference (RD) and the absolute risk difference (ARD) were calculated. Pooled continuous variables were reported as mean differences (MDs) and corresponding 95% CIs using the Mantel-Haenszel fixed effect method.

Forest plots were created for graphical representation of RRs and MDs. Heterogeneity was measured using the I^2^ statistic. Significant heterogeneity was considered present if the I^2^ statistic was >50%. We explored statistical heterogeneity using post-hoc sensitivity analyses. Subgroup analyses were planned according to gestational age (term vs late preterm infants), mode of delivery and type of suctioning device (bulb vs catheter).

Communication of the findings of the review was based on GRADE guidelines with wording decided by the ILCOR NLS Task Force through consensus.

## Results

### Literature search and study selection

**Fig. 1:**
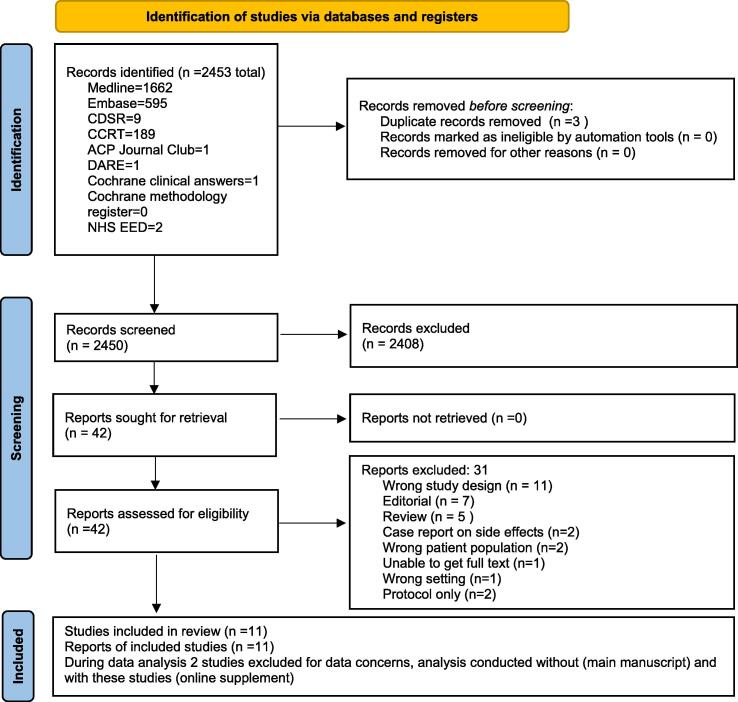
PRISMA flow diagram of study selection.

The search strategies identified 2453 unique records, for which titles and abstracts were screened, and 2411 studies were excluded (Fig. 1). From them, 42 full-text articles were assessed for eligibility and 11 were included in the final review.

## Study Characteristics

The SR included 9 RCTs^3^, ^4^, ^5^, ^9^, ^24^, ^25^, ^26^, ^27^, ^28^ and 2^14^, ^29^ observational studies enrolling a total of 1514 newborn infants (1096 in RCTs, 418 in observational studies) (Table 1). All the RCTs only recruited term newborn infants except for one^26^ that recruited newborn infants >35 weeks. One observational study^29^ recruited only term newborn infants whilst another^14^ recruited term and preterm newborns.

**Table 1:** Study Characteristics.

Study Year Country	Design	Eligibility	Enrolled (n)	Suction	No suction	Outcomes	Main Findings
Bancalari 2019 Chile	RCT	Term infants born by C-section	84	n = 42 Catheter tube size 8 introduced 6 cm Negative pressure < 30cmH2O Procedure 15 sec	n = 42 No suction Routine care; cleaning	Continuous readings of oxygen saturations and heart rate over the first 10 minutes of life and at 15,30 and 60 minutes	Mean ± SD SaO_2_ at 1 minute of life was 52.6 ± 7.6% (ONPS) vs 56.1 ± 10.8% (no ONPS) with no significant difference (p = 0.28). Mean ± SD HR at 1 minute of life was 137 ± 25 (no suction) 148 ± 13 (suction) (p = 0.02), but no difference was found in the subsequent minutes
Carrasco 1996 Uruguay	RCT	Singleton, term infants, cephalic vaginal delivery, no maternal/fetal pathological changes, no medication before/during labour	30	n = 15 Suction with catheter tube 3R polyethylene, first nasopharynx then nose no more 6 cm for 8 to 10 sec, negative pressure < 30cmH_2_O	n = 15 No suction	Continuous readings of oxygen saturations and heart rate over the first 20 minutes of life Minutes to 86% and 92% SaO_2_	The ONPS group had a significantly lower SaO_2_ between the first and the sixth minutes of life and took longer to reach 86% and 92% saturation.
Estol Uruguay	RCT	Singleton, term infants with no fetal/maternal morbidity Well baby Membranes intact or ruptured < 24 hours	40	n = 20	N = 20	Spirometric assessment at 10, 30 and 120 minutes	No significant differences between suction and no suction groups were seen for any of the parameters of respiratory mechanics.
Gungor 2005 Turkey	RCT	Term infants, vaginal delivery	140	n = 70 Catheter tube 8 Ch, polyethylene, negative pressure < 30cmH2O procedure 15 sec	n = 70 No suction or wipe away any visible matter	SaO_2_ measured minute-by minute from the first minute of life until 92% was reached. Apgar scores at one and five minutes Proportion of group that achieved 86% and 92% SaO_2_ by minute of life	The no suction group showed lower mean heart rates through the 3rd and 6th minutes and higher SaO_2_ values through the first 6 mins of life (p < 0.001). The maximum time to reach SaO_2_ of ≥92% (6 vs 11 min) and ≥86% (5 vs 8 min) were shorter in the no suction group (p < 0.001). HR and SaO_2_ is remarkably similar in the 2005 and 2006 Gungor studies.
Gungor 2006 Turkey	RCT	Term infants, caesarean section	140	n = 70 Catheter tube 8 Ch, polyethylene, negative pressure < 30cmH2O procedure 15 sec	n = 70 No suction or wipe away any visible matter	SaO_2_ measured minute-by minute from the first minute of life until 92% was reached. Apgar scores at one and five minutes Proportion of group that achieved 86% and 92% SaO_2_ by minute of life	Mean SaO_2_ values through 2nd to 6th min of life were significantly higher in the no suction group (p < 0.001). Maximum time to reach SaO_2_ of 92% (6 vs 11 min) and 86% (5 vs 8 min) were shorter with no ONPS. Mean HR was consistently and significantly lower with no ONPS during the first 6 mins except the second one. All neonates without suction had an Apgar score of 10 at five mins, while the mean ± SD for ONPS group was 9.34 ± 0.48 (p < 0.001).
Kelleher 2013 USA	RCT	Infants ≥35 weeks gestation	448	n = 242 suction mouth and nostrils with bulb syringe	n = 246 Gentle wiping externally over face, mouth and nose with towel	Primary outcome: respiratory rate (RR) in first 24 hours after birth	Mean RR in the first 24 hours were 51 (SD 8) breaths per min in the wipe group and 50 (6) breaths per min in the suction group (difference of means 1 breath per min, 95% CI –2 to 0, p < 0·001).
Modarres Nejad 2014 Iran	RCT	Term infants vaginal delivery	170	n = 85 Suction: < 15 sec after birth with polyethylene catheter Negative pressure < 30cmH_2_O	n = 85 No suction: was only to remove any visible material.	SaO_2_ measured minute-by minute from the first minute of life until 92% was reached. Apgar scores at 1 and 5 minutes	Maximum time to reach SaO_2_ of 92% was shorter in the no suction group. Mean SaO_2_ values from first to fifth min of life were similar in the two groups. No statistically significant differences in the mean of HR, RR and Apgar scores between the groups.
Takahashi 2009 Japan	RCT	Term, weight 2500–4000 g Apgar ≥8 at 1 and 5 mins vaginal delivery	26	n = 13	n = 13	SaO_2_ and heart rate documented every 30 seconds from five minutes of life until two hours later. Two outcomes were defined, time to reach SaO2 of ≥96% and time to HR of ≤ 160 bpm	There was no statistically significant difference in the time to stabilise SaO2 ≥96% or HR ≤ 160 bpm. Observations up to 10 minutes after birth, showed no statistically significant difference, but the non-suction group tended to stabilize both SpO2 and HR earlier than the suction group.
Waltman 2004 USA	RCT	Term infants, vaginal delivery	20	n = 10 Suction mouth and nose one time each with 2-ounce soft rubber bulb syringe or ear/ulcer syringe 1.5 inches deep, and finger pressure, when the head was delivered, and mouth and nose wiped with a towel if any visible matter	n = 10 No suction, mouth and nose wiped with a towel if any visible matter	Apgar scores, heart rates, and oxygen saturation levels in the first 20 minutes of life	Newborns receiving bulb suctioning had a lower heart rate (P = 0.042) during the first 20 minutes and a significantly higher SpO_2_ level (P = 0.005) by 15 minutes of age. Although statistically significant, these findings were not considered clinically significant because values remained within normal parameters. There were no statistically significant differences in Apgar scores between groups.
Konstantelos 2015 Germany	Obs	All newborns with a GA > 28 completed weeks were included Term & preterm subgroups analysed		115	231	Single-centre analysis of video-recorded delivery room management after c-section. Time point, duration, and frequency of suctioning in term and preterm newborns were analysed along with (heart rate (HR) and saturation values). Respiratory support (yes/no) reported	36/60 term infants needing respiratory support were suctioned 22/200 term infants without respiratory support were suctioned 56/71 preterm infants needing respiratory support were suctioned 1/15 preterm infants without respiratory support were suctioned Newborns were suctioned up to 14 times; total duration spent for suctioning was between 2 and 154 s. Suctioning before face mask application in 31% of the suctioned newborns requiring respiratory support. Term infants who did not require respiratory support showed significantly higher saturation values at 3, 5, 6, 7, 8, 9, and 10 min if they were not suctioned. No severe bradycardia (<60 bpm) Suctioning had no effect on HR and SaO_2_ in preterm infants but was associated with significantly higher HR in term infants requiring respiratory support.
Pocivalnik 2015	Obs	Term neonates after elective caesarean section	72	36 suction catheter	36 no suction	Heart rate (HR) and pre/post SaO_2_ ductal arterial oxygen saturation measured by pulse oximetry. Cerebral and pre/post ductal peripheral muscle tissue oxygenation were measured by near infrared spectroscopy during the first 15 min of life.	All neonates in both groups had normal Apgar scores (Apgar 9/10/10) and no events of apnoea or bradycardia induced by suctioning. SpO_2_ pre values were slightly lower in the suctioned group at 2 and 4 min after birth. Suctioning had no main and interaction effect on HR, SpO_2_ post ductal, rSO_2_brain, rSO_2_perpheral muscle tissue pre and post ductal in the first 15 min after birth.

SD: standard deviation, ONPS: oronasopharyngeal suction, RCT: randomised controlled trial, HR: heart rate, SaO_2_ arterial oxygen saturation, RR: respiratory rate, SpO_2_ pulse oximetry, rSO_2_: tissue oxygenation.

For two of the RCTs^3^, ^4^ enrolling 280 participants, the task force had concerns about the reliability of the oxygen saturation and heart rate data. The reported standard deviations were unusually small in comparison to other published studies and the data in each study were remarkably similar. The author was contacted to provide clarification; however, at the time of publication the task force had not received a reply. Therefore, the results of these studies have been excluded from the meta-analysis. Exclusion of these studies did not change the conclusion of this systematic review but in the interests of transparency, analyses were repeated including these two studies and the results are shown in an online supplement.

### Risk of bias

RoB was increased for all studies because blinding of those performing the intervention to group assignment was not considered feasible (Table 2). Some concerns about selective reporting of outcomes were present for two studies.

**Table 2:** Certainty of evidence by outcome, relative risks and anticipated absolute effects.

	Certainty assessment				Summary of findings				
No. of studies Participants	RoB	Inconsistency	Indirectness	Imprecision	Number of patients		Effect		Certainty
					suctioning	No suctioning	Relative 95% CI	Absolute 95%CI	
**Receipt of Assisted ventilation (primary outcome)**									
3 742	very serious	serious	very serious	very serious	17/369 (4.6%)	24/373 (6.4%)	RR 0.72 (0.4 to 1.31)	18 fewer per 1000 (39 fewer to 20 more)	Very Low
**Advanced Resuscitation and stabilisation interventions (intubation, chest compressions, epinephrine (adrenaline) in delivery room**									
3 742	very serious	serious	very serious	very serious	17/369 (4.6%)	24/373 (6.4%)	RR 0.72 (0.4 to 1.31)	18 fewer per 1000 (39 fewer to 20 more)	Very Low
**Saturations at 5 minutes**									
3 280	serious	serious	very serious	not serious	140	140		Saturation % MD 0.26 lower (1.77 lower to 1.26 higher)	Very Low
**Saturations at 9 minutes**									
3	very serious	serious	very serious	not serious	140	140		Saturation % MD 1.52 lower (2.69 lower to 0.35 higher)	Very Low
**Saturations at 10 minutes**									
2	serious	serious	very serious	not serious	55	55		Saturation % MD 0.14 lower (1.17 lower to 0.89 higher)	Very Low
**Respiratory rate > 60 in first 24 hours**									
1	not serious	not serious	serious	not serious	112/246 (46.3)	113/246 (45.9%)	RR 0.99 (0.82 to 1.2)	5 fewer per 1000 (83 fewer to 92 more)	Moderate
**Heart rate at 5 minutes**									
1 84	serious	not serious	very serious	Not serious	42	42		MD −1.00 (-7.96 lower to 5.96 higher)	Very Low
**Unanticipated admission to NNU**									
1 448	serious	not serious	serious	very serious	30/242 (12.4%)	45/246 (18.6%)	RR 1.50 (0.96 to 2.3)	91 more per 1000 (7 fewer to 238 more)	Very Low

No.: number, RoB: risk of bias, CI: confidence interval, NNU: Neonatal Unit.

### Certainty of evidence

Evidence for the primary and all but one of the secondary outcomes was rated as low or very low certainty because of high RoB and indirectness (Table 2). As the studies predominantly recruited healthy term newborn infants, they were downgraded for indirectness for all outcomes because they were not considered representative of all newborn infants, including those at high risk of need for assisted ventilation or other adverse outcomes.

## Outcomes

***Primary outcome*** - Assisted ventilation: Three RCTS^24^, ^26^, ^27^, including 702 participants found that for suctioning compared to no suctioning, clinical benefit or harm could not be excluded (RR 0.72; 95% CI 0.40, 1.31 p = 0.28; absolute risk difference (ARD) 18 fewer per 1000 95% CI, 39 fewer to 20 more per 1000). Two of these RCTs^24^, ^27^ recruited healthy infants and reported assisted ventilation was not required so the event rate was zero in both groups. Evidence was of very low certainty (downgraded for very serious risk of bias, serious inconsistency, very serious indirectness and very serious imprecision).

### Secondary outcomes

**Advanced resuscitation and stabilization interventions (intubation, chest compressions/epinephrine (adrenaline) in DR)** Very low certainty evidence from three RCTS^24^, ^26^, ^27^ including 702 participants found that for suctioning vs no suctioning, clinical benefit or harm could not be excluded (RR 0.72; 95% CI, 0.40, 1.31p = 0.28; ARD 18 fewer per 1000 95% CI, 39 fewer to 20 more patients per 1000). Two of these RCTs^24^, ^27^ recruited healthy infants and reported advanced resuscitation was not required so the event rate was zero in both groups. Evidence was downgraded for very serious risk of bias, serious inconsistency, very serious indirectness and very serious imprecision.

***Receipt and duration of oxygen supplementation***: Two RCTs^24^, ^27^ included 254 healthy term infants and reported all newborns were born in good clinical condition and did not need supplemental oxygen. Clinical benefit or harm could not be excluded as the event rate was zero in both groups so a relative risk could not be calculated.

**Oxygenation outcomes (** Table 3 **)**

**Table 3:** Oxygen saturation outcomes infants receiving oronasopharyngeal suctioning vs no suctioning.

Variable		Result (suctioning vs not suctioning)	Comments
Oxygen saturations	At 1 minute	2RCTS, 254 participants clinical benefit or harm could not be excluded MD −0.67% (95%CI, −2.62 to 1.27%)	
	5 minutes	3RCTS, 280 participants clinical benefit or harm could not be excluded MD −0.26% (95%CI, −1.77 to 1.26%)	
	9 minutes	3 RCTS, 280 participants possible harm MD −1.52% (95% CI, −2.69 to −0.35%)	statistically significant but of unclear clinical significance
	10 minutes	2 RCTs, 110participants clinical benefit or harm could not be excluded MD −0.14 (95%CI, −1.17, 0.89)]	
Oxygen saturations over first 10 minutes	**Bancalari**: non-significantly lower SaO_2_ in group with suction over 1st 4 minutes, no difference from 4-10 minutes **Carrasco**: average SaO_2_ was significantly lower (p < 0.05, one tail) in the suctioned group from 1 to 6 minutes **Konstantelos**: lower SaO_2_ over first 10 minutes with suctioning (p < 0.05) **Modarres:** lower SaO_2_ with suctioning (<p < 0.002) at 9 minutes **Pocivalnik**: lower SaO_2_ with suctioning (p < 0.05) at 2 and 4 minutes not at other times **Waltman**: lower saturations at 5 minutes, higher at 10 minutes in suctioned group, both findings not significant		Both excluded Gungor studies showed lower SaO_2_ over first 6 minutes (p < 0.001) Some studies displayed data graphical rather than numerically precluding meta-analysis or calculating MD (95%CI)
Proportion reached 92% saturation	10 minutes	**Modarres:** suctioned 90.6% not suctioned 100%	
Time in minutes to reach	86% SaO_2_ 92% SaO_2_	**Carrasco**: 8.2 ± 3.3 vs 5.0 +/-1.2 (suctioned vs not suctioned) **Carrasco**: 10.2 ± 3.3 vs 6.8 +/-1.8 (suctioned vs not suctioned)	Carrasco - time to reach 86% and 92% saturations significantly shorter in the non-suctioned group (p < 0.05)

Both excluded Gungor studies showed maximum time to SaO2 ≥92% (6 vs 11 min) and ≥86% (5 vs 8 min) were shorter in the no suction group (P < 0.001).

MD: mean difference, CI: confidence interval, SD: standard deviation, RCT: randomised controlled trial, SaO_2_ arterial oxygen saturation.

**Fig. 2:**
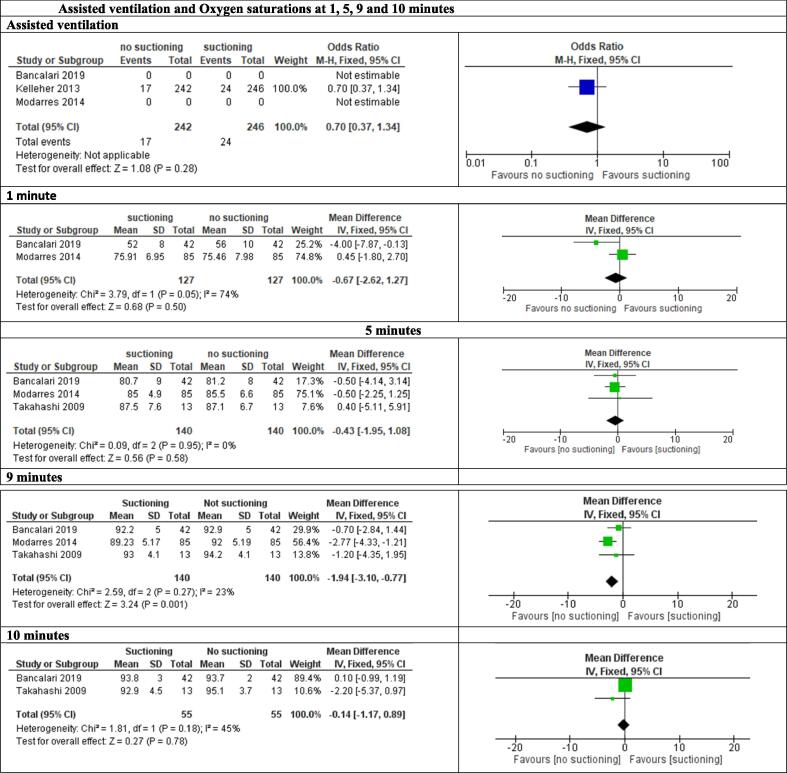
Assisted ventilation and Oxygen saturations at 1, 5, 9 and 10 minutes.

**Oxygen saturations at 1, 5, 9 and 10 minutes (** Fig. 2 **)**

Very low certainty evidence for oxygen saturations at one^24^, ^27^ five^24^, ^27^, ^28^ and ten^24^, ^28^ minutes could not exclude benefit or harm. Data at 9 minutes from 3 RCTS^24^, ^27^, ^28^ including 280 participants, suggested possible harm for suctioning vs no suctioning (MD −1.52% 95% CI, −2.69 to −0.35%). This finding was statistically significant but of unclear clinical significance. Evidence was downgraded for very serious risk of bias, serious inconsistency and very serious indirectness.

**Oxygen saturations over the first 10 minutes from birth:** Data were presented in different ways in different studies, precluding a comprehensive meta-analysis of all studies that reported data on this outcome. Two RCTs^9^, ^27^ (200 participants) and 2 observational studies^14^, ^29^ (418 participants) found lower oxygen saturations in those receiving suctioning within first 10 minutes, while two other RCTs^5^, ^24^ did not find significant differences. All evidence was of very low certainty.

**Time to reach target oxygen saturations of 86% or 92%:** One RCT^9^ found time to reach 86% SaO_2_ and two RCTs^9^, ^27^ found time to 92% SaO_2_ was quicker in the non-suctioned group. One RC showed that 90.6% of those suctioned had achieved 92% saturations at 10 minutes vs 100% of those not suctioned. The oxygenation targets were those selected by the authors.

**Other oxygenation outcomes:** One prospective observational study^14^ including 346 participants reported 1 episode of severe desaturation to <75% following suctioning. One prospective observational study^29^ enrolled 138 infants born at term by elective caesarean section to examine cerebral and peripheral muscle tissue oxygenation. Between groups of 36 infants who received oropharyngeal suctioning and 36 controls, there was no difference in heart rate, oxygen saturations, cerebral and peripheral muscle tissue oxygenation.

**Respiratory rate >60 in the first 24 hours:** Moderate certainty evidence from one RCT with 488 participants (not restricted to healthy infants and including those ≥35 weeks’ gestation), showed clinical benefit or harm could not be excluded (RR 0.99; 95% CI, 0.82, 1.20); ARD 5 fewer per 1000 with those receiving suctioning vs no suctioning (95% CI, 83 fewer to 92 more per 1000 patients receiving suctioning).

**Heart rate at 5 minutes:** Very low certainty evidence from one RCT^24^ including 84 participants found clinical benefit or harm could not be excluded [MD −1.00 (95%CI, −7.96, 5.96)] however both groups had a heart rate in the normal range and no bradycardias were reported in either group. Evidence was downgraded for inconsistency and indirectness.

**Apgar scores:** Insufficient data on the secondary outcome of low Apgar scores (<7) was available for analysis. For the outcome of Apgar score of 10 at 5 minutes very low certainty evidence from one RCT^27^ including 170 participants showed clinical benefit or harm could not be excluded [MD 1.00 (0.98, 1.02)].

**Unanticipated admission to the NICU:** Very low certainty evidence from one RCT^26^ including 448 infants of ≥35 weeks’ gestation showed clinical benefit or harm cannot be excluded (Relative risk [RR], 1.50; 95% CI, 0.96, 2.30) ARD 91 more per 1000 with no suctioning vs suctioning (95% CI, 8 fewer to 238 more per 1000 patient receiving no suctioning). Evidence was downgraded for RoB, inconsistency and indirectness.

**Other secondary outcomes:** Insufficient data were available to be able to report on the important secondary outcomes of soft tissue injury, infection and bradycardia.

### Subgroup analyses

**Gestational age:** Insufficient data were available for this subgroup analysis. Only one prospective observational study^14^ and one RCT^26^ included both preterm and term infants although most babies in both studies were born at term.

**Vaginal vs Caesarean section:** insufficient data were available for a subgroup analysis of the following outcomes: receipt of assisted ventilation, advanced resuscitation, receipt of supplemental oxygen, unanticipated NICU admission.

For the outcome of oxygen saturations at 5 minutes there was a difference favouring no suctioning in both vaginal delivery and caesarean section subgroups with high heterogeneity within subgroups (I^2^ = 97%) and evidence of an interaction by delivery type (test for subgroup differences 0.03) also with high heterogeneity between subgroups (I^2^ = 78.6%). Given the very high heterogeneity, despite almost identical results in two studiesp^3^, ^4^ a sensitivity analysis was carried out. With the two Gungor studies^3^, ^4^ removed from both subgroups there was no difference in saturations in either subgroup with no interaction (p = 0.86) and heterogeneity reduced (I^2^ = 0%).

Among the two methodologically identical RCTs by Gungor^3^, ^4^ one studied vaginally born infants and the other those born by caesarean section, each included 140 participants and found identical times to achieve saturations of 86% or 92%.

### Suction device used (Bulb vs Catheter Suction)

Two RCTs^5^, ^26^ studied infants receiving bulb suction vs no suction or wiping but no studies compared bulb suction to catheter suction.

## Discussion

This systematic review (SR) analysed 9 RCTs^3^, ^4^, ^5^, ^9^, ^24^, ^25^, ^26^, ^27^, ^28^ and 2 prospective observational studies^14^, ^29^ all of which noted that suctioning of clear amniotic fluid from the mouth and/or nose has been a common or routine historical practice in many parts of the world. The procedure is still used frequently, and suctioning can take a long time,^14^ thereby potentially delaying the start of necessary critical interventions such as positive pressure ventilation. Most international guidelines recommend that if aeration of the lungs is difficult and airway obstruction is suspected then positioning to improve airway patency and if necessary, suctioning should be performed.

This systematic review found no evidence of benefit of suctioning the upper airway (compared to no suctioning) although evidence was very low certainty. Several studies reported lower oxygen saturations in infants receiving suctioning. However, combining the data for a meta-analysis was not possible due to differences in the presentation of data in the included studies. Some studies reported continuous measurements over time, others reported time to achieve a certain saturation.

Two RCTs^3^, ^4^ enrolling 280 participants, were originally selected for inclusion but were excluded post-hoc. The studies, which enrolled distinct groups of newborn infants (one enrolled infants born by caesarean section and the other, vaginal births) reported almost identical results for oxygen saturation levels, with much smaller standard deviations than those seen in other studies. Because a data reporting error was considered possible, a decision was made to omit the studies from the review. For transparency, analyses including them are shown in Appendix A. Their inclusion would have made little difference to the overall findings of the systematic review.

There are case reports in the literature of rare potential side effects of upper airway suctioning including cardiac arrest in one case.^7^ The studies included in the review did not report any instances of severe bradycardia, but they are of insufficient size to assess low frequency adverse events. In the absence of evidence of benefit, it seems unjustified to expose large numbers of newborn infants to any risk of harm by using upper airway suctioning.

The review could not exclude the possibility that there are subgroups of newborn infants who could benefit from upper airway suctioning. The focus of this review was infants with clear amniotic fluid, so the results cannot be considered to apply to those with blood clots, meconium or other particulate material in the amniotic fluid. The included studies included mostly healthy term infants, limiting the generalisability to preterm babies or those requiring resuscitation. We found no studies that targeted recruitment of depressed or very preterm infants.

Strengths of this review include that it was conducted rigorously and in accord with a pre-registered protocol that was developed in collaboration with the combined expert opinion of the ILCOR NLS Task Force. It used a search strategy developed by an expert information specialist and was performed in adherence with established guidelines for systematic reviews. Limitations include the difficulties of obtaining additional information from authors and the differences in presentation of study results in the included studies, which precluded some of the intended meta-analyses, as well as pre-planned subgroup analyses. This may have prevented recognition of important subgroups of infants in whom the balance of risks and benefits differs.

## Conclusion

This systematic review found no evidence of benefit for routine suctioning of clear amniotic fluid, compared to no suctioning, although the evidence is of low to very low certainty. There was also very low certainty evidence of a temporary adverse effect on oxygen saturation levels, of uncertain clinical significance. The review supports current guidelines which advise against routine suctioning of the upper airway in infants with clear amniotic fluid.

## Contributor’s statement

Drs. Fawke, Wyllie, Udaeta, Rüdiger and Ersdal prepared the protocol, screened studies, completed full text reviews, abstracted data, completed risk-of-bias and GRADE evaluations, completed the analysis, and prepared the manuscript.

Mary-Doug Wright developed the search strategy with the review group and conducted the initial and updated literature searches.

Drs. Liley, Weiner, Wyckoff, and Rabi reviewed the protocol, abstracted data, reviewed the analysis and edited the manuscript.

The review group included Drs Fawke, Wyllie, Udaeta, Rüdiger and Ersdal.

All authors approved the final manuscript as submitted and agree to be accountable for all aspects of the work.

## Publication statement

This systematic review and meta-analysis was performed under the umbrella of the 2022 Neonatal Consensus on Science with Treatment Recommendations (CoSTR) and evidence to Decision (EtD) framework. Whilst a summary of this systematic review and meta-analysis will be included in the 2022 CoSTR paper, the submitted systematic review and meta-analysis is a more detailed version which includes all related data, figures and tables. It has not been published previously and the manuscript is not under consideration elsewhere.

## Conflict of interest disclosures

The authors have no conflicts of interest relevant to this article to disclose. 

## Funding/support

The International Liaison Committee on Resuscitation provided support that included access to software platforms, an information specialist and teleconferencing.

## Figures and Tables

**Table:** 

1	exp Infant, Newborn/or premature birth/or newborn/or prematurity/	1,298,648
2	(newborn* or new-born* or infant* or neonat* or neo-nat* or newly born* or delivery room* or prematur* or preterm or postmatur* or pre-matur* or pre-term or post-matur* or prematuritas or postnatal or post-natal).ti,ab,kw,kf.	2,151,537
3	1 or 2 [NEWBORN]	2,657,669
4	Suction/	24,930
5	(suction* or ONPS or (mechanical* adj4 aspirat*) or (airway* adj4 (clear* or aspirat*)) or “nasopharyngeal stimulation” or “oronasopharyngeal suction” or “naso-pharyngeal stimulation” or “oro-nasopharyngeal suction” or “oronaso-pharyngeal suction” or “oro-naso-pharyngeal suction”).ti,ab,kw,kf.	58,390
6	4 or 5 [SUCTION]	69,884
7	3 and 6 [NEWBORN + SUCTION]	5664
8	(Animals/or “Animal Experimentation”/or “Models, Animal”/or “Disease Models, Animal”/) not (Humans/or “Human Experimentation”/)	8,672,741
9	7 not 8 [ANIMAL ONLY REMOVED]	5338
10	(comment or editorial or “newspaper article” or news or note or lecture).pt.	3,230,033
11	(letter not (letter and randomized controlled trial)).pt.	2,406,509
12	9 not (10 or 11) [OPINION PIECES REMOVED]	5110
13	“case reports”.pt.	2,274,513
14	12 not 13 [CASE REPORTS REMOVED]	4770
15	(conference or “conference abstract” or “conference review” or congresses).pt.	5,202,400
16	14 not 15 [CONFERENCES REMOVED]	4067
17	Trachea/	65,388
18	trachea*.ti,ab,kw,kf.	153,962
19	17 or 18 [TRACHEA]	172,898
20	(nasopharyngeal or oronasopharyngeal or naso-pharyngeal or oro-nasopharyngeal or oronaso-pharyngeal or oro-naso-pharyngeal).ti,ab,kw,kf.	82,327
21	19 and 20 [STUDIES WITH BOTH TRACHEA AND NASOPHARYNGEAL]	895
22	19 not 21 [TRACHEA ONLY]	172,003
23	16 not 22 [TRACHEA ONLY REMOVED] Embase <1974 to 2022 June 16> Ovid MEDLINE(R) and Epub Ahead of Print, In-Process, In-Data-Review & Other Non-Indexed Citations and Daily <1946 to June 16, 2022>	3440 1775 1665

24	remove duplicates from 23 Embase <1974 to 2022 June 16> Ovid MEDLINE(R) and Epub Ahead of Print, In-Process, In-Data-Review & Other Non-Indexed Citations and Daily <1946 to June 16, 2022>	2292 631 1661

**Table:** 

#1	[mh “Infant, Newborn”]	17,498
#2	(newborn* OR new-born* OR infant* OR neonat* OR neo-nat* OR newly NEXT born* OR delivery NEXT room* OR prematur* OR preterm OR postmatur* OR pre-matur* OR pre-term OR post-matur* OR prematuritas OR postnatal OR post-natal):ti,ab,kw	95,011
#3	#1 OR #2	95,011
#4	[mh Suction]	953
#5	(suction* OR ONPS OR (mechanical* AND aspirat*) OR (airway* NEXT (clear* OR aspirat*)) OR ((clear* OR aspirat*) NEXT airway*) OR “nasopharyngeal stimulation” OR “oronasopharyngeal suction” OR “naso-pharyngeal stimulation” OR “oro-nasopharyngeal suction” OR “oronaso-pharyngeal suction” OR “oro-naso-pharyngeal suction”):ti,ab,kw	5926
#6	#4 OR #5	5926
#7	#3 AND #6	654
#8	[mh Trachea]	393
#9	trachea*:ti,ab,kw	8327
#10	#8 OR #9	8327
#11	(nasopharyngeal OR oronasopharyngeal OR naso-pharyngeal OR oro-nasopharyngeal OR oronaso-pharyngeal OR oro-naso-pharyngeal):ti,ab,kw	3457
#12	#10 AND #11	62
#13	#10 NOT #12	8265
#14	#7 NOT #13	523
#15	([mh ^Animals] OR [mh ^“Animal Experimentation”] OR [mh ^“Models, Animal”] OR [mh ^“Disease Models, Animal”]) not ([mh ^Humans] OR [mh ^“Human Experimentation”])	4
#16	#14 NOT #15	523
#17	(comment OR editorial OR “newspaper article” OR news OR note OR lecture):pt	15,015
#18	(letter NOT (letter AND randomized controlled trial)):pt	7605
#19	#16 NOT (#17 OR #18)	520
#20	“case reports”:pt	1649
#21	#19 NOT #20	519
#22	(conference OR “conference abstract” OR “conference review” OR congresses):pt	198,887
#23	#21 NOT #22	483
#24	#21 NOT #22 with Cochrane Library publication date Between Aug 2021 and Jun 2022 CDSR: 1; CCRCT (Trials): 29	30

## Appendix A Definitions used in this systematic review

**Advanced resuscitation and stabilization interventions:** intubation, chest compressions/epinephrine (adrenaline) in the delivery room.

**Adverse effects of intervention**: e.g., apnoea, bradycardia, oxygen saturations, heart rate, injury, infection, low Apgar scores, dysrhythmia.

**Assisted Ventilation:** receipt of positive pressure ventilatory support including Continuous Positive Airways Pressure (CPAP).

**Bradycardia** heart rate less than 100 beats per minute for 10 seconds or longer during or immediately (<20 seconds) following suctioning.

**Cardiac dysrhythmias:** any variation of normal cardiac rhythm or rate of heartbeat during or immediately (<20 seconds) following suctioning.

**Clear amniotic fluid:** clear or slightly yellowish liquid that surrounds the unborn baby (fetus) during pregnancy. It is contained in the amniotic sac (Jacobsen 2018). It can sometimes be blood stained during delivery.

**Episodes of apnoea** cessation of breathing for more than 20 seconds or a shorter pause associated with bradycardia or cyanosis (AAP 2003 914) during initial oro/naso/pharyngeal suctioning or immediately (within 20 seconds) following initial mouth or nose (Oro/nasopharyngeal suctioning) or both.

**Initial Suctioning:** Suctioning of the mouth or nose (Oro/nasopharyngeal suctioning) as an initial action prior to any other airway and breathing manoeuvres (excluding head positioning).

**Newly born**: first hour of life.

**Suction of the mouth or nose (Oro/nasopharyngeal suction)** is a method used to clear secretions from the oropharynx or nasopharynx, or both, through the application of negative pressure via a suction catheter or bulb syringe. {Waltman 2004 32}.

**Unexpected admission to NICU:** >34 weeks gestation infant admitted to NICU but not as a result of a protocol that is based purely on birthweight or gestation (as opposed to clinical condition).

In addition to the authors (JF, JW, EU, MR, HE MHW, HGL, YR, GMW), the following ILCOR NLS Task Force members provided input on the review protocol, the interpretation of the results, and the article as experts in neonatal resuscitation: Dr. Daniela T. Costa-Nobre, Federal University of São Paulo, São Paulo, Brazil; Dr. Peter G. Davis, The Royal Women’s Hospital, Victoria, Australia; Dr. Maria F. de Almeida, Federal University of São Paulo, São Paulo, Brazil; Dr. Walid El Naggar, Dalhousie University, Halifax, Nova Scotia, Canada; Dr. Jorge G. Fabres, Universidad Catolica de Chile, Santiago, Chile; Dr. Elizabeth E. Foglia, University of Pennsylvania, Philadelphia, Pennsylvania; Dr. Ruth Guinsburg, Federal University of São Paulo, São Paulo, Brazil; Dr. Tetsuya Isayama, National Center for Child Health and Development, Tokyo, Japan; Dr. Vishal S. Kapadia, University of Texas Southwestern Medical Center, Dallas, Texas; Dr. Mandira D. Kawakami, Federal University of São Paulo, São Paulo, Brazil; Dr. Han-Suk Kim, College of Medicine, Seoul National University, Seoul, Korea; Dr. Henry C. Lee, Stanford University School of Medicine, Palo Alto, California; Dr. R. John Madar, University Hospitals Plymouth NHS Trust, Plymouth, United Kingdom; Dr. Christopher J.D. McKinlay Kidz First Neonatal Care, Auckland, New Zealand, Dr. Firdose L. Nakwa, University of Witwatersrand, Johannesburg, South Africa; Dr. Jeffrey M. Perlman, Weill Cornell Medical College, Cornell University, New York, New York; Dr. Charles C. Roehr, Oxford University Hospitals, National Health Service Foundation Trust, United Kingdom; Dr. Georg M. Schmölzer University of Alberta, Canada, Dr. Takahiro Sugiura, Toyohashi Municipal Hospital, Toyohashi, Aichi, Japan; Dr. Daniele Trevisanuto, University of Padua, Padua, Italy.

## Appendix B Search strategy

### Summary

Records from database searches were downloaded and imported into an EndNote database to facilitate removal of duplicates and screening. Final database searches were conducted September 11, 2021. Update searches were conducted June 17, 2022.

**Embase 1974 to 2022 June 16, Ovid MEDLINE(R) and Epub Ahead of Print, In-Process, In-Data-Review & Other Non-Indexed Citations and Daily 1946 to June 16, 2022**

**Cochrane Library via Wiley Online**

CDSR Issue 6 of 12, June 2022.

CCRCT Issue 5 of 12, May 2022.
