# Supplementary material for: Suctioning of clear amniotic fluid at birth: A systematic review
Source: Resusc Plus. 2022 Sep 17;12:100298. doi: 10.1016/j.resplu.2022.100298 (PMC9490170; doi:10.1016/j.resplu.2022.100298)
Supplement: Supplementary data 1 [file mmc1.docx]

**Online Supplement**

This online supplement shows the analyses with the Gungor 2005^3^ and Gungor 2006^4^ RCTs included. Each had 140 healthy term participants 70 who received suctioning and 70 who did not. One recruited newborns born by vaginal delivery and one newborns born by caesarean section. These were not included in the main analysis as questions around the unusually similar saturation and heart rate data in the two studies could not be resolved (table 4).

**Primary outcome** **- Assisted ventilation:** Very low certainty evidence from five RCTS^3,4,24,25,26^ including 1022 participants found that for suctioning compared to no suctioning, clinical benefit or harm could not be excluded (Relative Risk) RR 0.72; 95% CI 0.40, 1.31 p=0.28; absolute risk difference (ARD) 13 fewer per 1000 95% CI, 28 fewer to 15 more per 1000). Evidence was downgraded for very serious risk of bias, serious inconsistency, very serious indirectness and very serious imprecision.

**Secondary outcomes**

**Advanced resuscitation and stabilization interventions**: Very low certainty evidence from five RCTS^3,4,24,25,26^ including 1022 participants found that for suctioning vs. no suctioning, clinical benefit or harm could not be excluded (RR 0.72; 95% CI, 0.40, 1.31 p=0.28; ARD 13 fewer per 1000 95% CI, 28 fewer to 15 more patients per 1000). Evidence was of very low certainty (downgraded for very serious risk of bias, serious inconsistency, very serious indirectness and very serious imprecision).

***Receipt and duration of oxygen supplementation***: Four RCTs^3,4,24,26^ included 534 healthy term infants and reported all newborns were born in good clinical condition and did not need supplemental oxygen. Clinical benefit or harm could not be excluded as the event rate was zero in both groups so a relative risk could not be calculated**.**

**Oxygen saturations (Table 3)**

**At 1 minute:** Very low certainty evidence including 4 RCTs^3,4, 24,27^ with 534 participants found for suctioning vs. no suctioning found possible benefit (mean difference [MD] -0.65% (95%CI, 0.45 to 0.65%)), although this was of unclear clinical significance.

**At 5 minutes:** Very low certainty evidence including 5 RCTs^3,4,24,27,28^ with 560 participants found for suctioning vs. no suctioning found possible harm (mean difference [MD] -9.10% (95%CI, -9.52 to -8.67%)).

**Oxygen saturations over the first 10 minutes from birth:** The two Gungor RCTs^3,4^ showed lower SaO_2_ over first 6 minutes (p<0.001). They also showed maximum time to SaO2 ≥ 92% (6 vs. 11 min) and ≥ 86% (5 vs. 8 min) were shorter in the no suction group (P < 0.001). All 140 infants with no suctioning achieved oxygen saturations of 86% by 5 minutes and 92% by 6 minutes. In contrast, only 2.9% of 140 infants receiving suctioning achieved saturations of 86% by 5 minutes and none achieved saturations of 92% by 6 minutes. Evidence was of very low certainty.

| **Figure 3 Oxygen saturation at 1 and 5 minutes including Gungor studies** | |
| --- | --- |
| **1 minute** | |
| 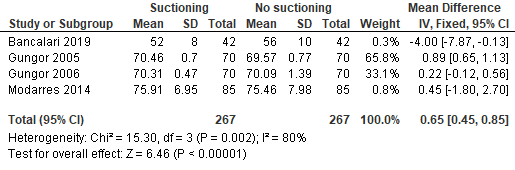 | 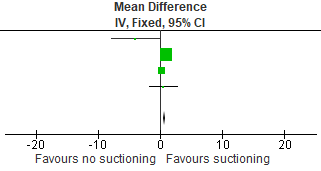 |
| **5 minutes** |  |
| 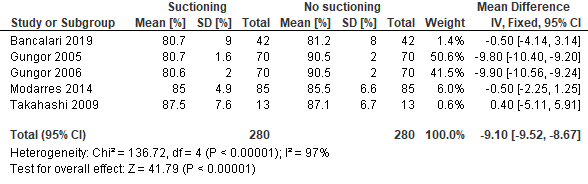 | 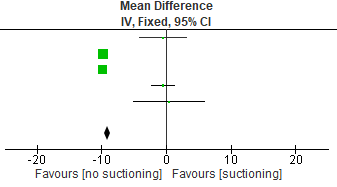 |

| **Table 4: Oxygen and Heart Rate data in the Gungor 2005^3^ and Gungor 2006^4^ studies** | | | | | | | | |
| --- | --- | --- | --- | --- | --- | --- | --- | --- |
|  | **Oxygen saturations** | | | | **Heart Rate** | | | |
| **Study** | **Gungor 2005** | | **Gungor 2006** | | **Gungor 2005** | | **Gungor 2006** | |
| **Group** | **No suction** | **Suction** | **No suction** | **Suction** | **No suction** | **Suction** | **No suction** | **Suction** |
| **Time** |  |  |  |  |  |  |  |  |
| 1 | 69.57+/-0.70 | 70.46 +/- 0.70 | 70.09 +/-1.39 | 70.31 +/-0.47 | 133.91 +/-7.87 | 136.60 +/-4.09 | 132.84 +/-10.71 | 137.36 +/-3.38 |
| 2 | 76.49 +/-2.77 | 72.86 +/-0.97 | 76.89 +/-3.48 | 72.47 +/-0.68 | 129.91 +/-6.86 | 131.51 +/-4.18 | 129.23 +/-9.95 | 132.63 +/-4.58 |
| 3 | 84.66+/-2.11 | 74.86+/-0.84 | 84.29 +/-2.68 | 75.04 +/-0.95 | 127.31+/-6.80 | 131.74 +/-4.40 | 127.40 +/-8.75 | 131.86 +/-5.54 |
| 4 | 86.89 +/- 2.72 | 77.60 +/-1.30 | 87.43 +/-3.09 | 77.64 +/-1.39 | 128.63 +/-7.79 | 132.94 +/-4.01 | 127.83 +/-8.90 | 132.70 +/-5.50 |
| 5 | 90.51 +/-2.03 | 80.71 +/-1.63 | 90.54 +/-2.00 | 80.60 +/-1.84 | 129.37 +/-5.62 | 134.00 +/-3.74 | 128.97 +/-6.44 | 134.54 +/-4.84 |
| 6 | 92.06 +/-0.23 | 83.37 +/-1.69 | 92.04 +/-020 | 80.60 +/-1.84 | 127.97 +/-4.40 | 135.06 +/-4.42 | 127.14 +/-6.07 | 134.87 +/-5.20 |
| 7 | - | 85.80 +/-1.10 | - | 83.36 +/-2.09 | - | 135.14 +/-5.08 | - | 135.90 +/-5.31 |
| 8 | - | 88.74 +/-1.39 | - | 85.83 +/-1.81 | - | 134.17 +/-3.80 | - | 135.31 +/-4.55 |
| 9 | - | 90.86 +/-1.16 | - | 88.64 +/-1.57 | - | 135.03 +/-2.77 | - | 135.47 +/-3.22 |
| 10 | - | 91.89 +/-0.40 | - | 90.80 +/-1.20 | - | 133.86 +/-2.31 | - | 134.80 +/-2.56 |
| 11 | - | 93..02 +/-1.23 | - | 91.96 +/-0.36 | - | - | - | - |

**Apgar scores:** For the outcome of achieving an Apgar score of 10 at 5 minutes very low certainty evidence from three RCTs^3,4,27^ including 450 participants showed possible harm (Relative risk [RR], 0.63; 95% CI, 0.57, 0.70) ARD (suctioning vs. no suctioning) 370 fewer (95% CI, 430 fewer to 300 fewer) per 1000 patients receiving suctioning). This finding was statistically significant but of unclear clinical significance.
